# Corrigendum to "New information of the biodiversity of the nymphalid family (Insecta, Lepidoptera, Nymphalidae) species collected in Romania’s fauna between 1887 and 1984"

**DOI:** 10.3897/BDJ.11.e103101

**Published:** 2023-07-03

**Authors:** Cristina Stancă-Moise, George Moise, Tom Brereton, Mirela-Aurora Stanciu

**Affiliations:** 1 "Lucian Blaga" University of Sibiu, Sibiu, Romania "Lucian Blaga" University of Sibiu Sibiu Romania; 2 Butterfly Conservation, Wareham, Dorset, United Kingdom Butterfly Conservation Wareham, Dorset United Kingdom; 3 Faculty of Agricultural Sciences, Food Industry and Environmental Protection, Sibiu, Romania Faculty of Agricultural Sciences, Food Industry and Environmental Protection Sibiu Romania

## Note

After the publication, we were notified of errors in the identification of some species from the presented historical collections (Stancă-Moise et al. 2023).

The updated names of the species in the collections should also be taken into account in Tables 1, 2, 3, 4, 5 and 6 from the article, which describe the results of the centralised data after the inventory of the collections (Daniel Czekelius (DC), Eugen Worell (EW), Viktor Weindel (VW), Rolf Weirauch (RW), Heinrich Hann von Hannenheim (HH), Eckbert Schneider (ES)).

We have revised all obsolete names to the current accepted name and cross-reference data with the current name and the name as originally used in the catalogue below.

Nymphalidae collected in Romania’s fauna between 1887 and 1984 (Table 1).

## Figures and Tables

**Table 1.** The names of the species in the collections and actual taxon.

**The names of the species in the collections (from article)**	**Taxon**
*Libytheaceltis* (Laicharting, 1782)	***Libytheaceltis* (Laicharting, 1782)**
*Argynnispaphia* (Linnaeus, 1758)	***Argynnispaphia* (Linnaeus, 1758)**
Argynnispaphiaf.valensina [Esper, 1798]	Current name: ***Argynnispaphia* (Linnaeus, 1758)**
*Argynnispandora* ([Denis & Schiffermüller], 1775)	***Argynnispandora* ([Denis & Schiffermüller], 1775)**
Argynnispandorasubsp.dacica Hormuzaki, 1892	Current name: ***Argynnispandora* ([Denis & Schiffermüller], 1775)**
Argynnis (Speyeria) aglaja (Linnaeus, 1758)	Current name: ***Speyeriaaglaja* (Linnaeus, 1758)**
Argynnis (Fabriciana) adippe ([Denis & Schiffermüller], 1775)	Current name: ***Fabricianaadippe* ([Denis & Schiffermüller], 1775)**
Argynnis (Fabriciana) niobe (Linnaeus, 1758)	Current name: ***Fabriciananiobe* (Linnaeus, 1758)**
Argynnis (Fabriciana) niobe f. cleodoxa Esper 1789	Current name: ***Fabriciananiobe* (Linnaeus, 1758)**
Argynnis (Fabriciana) niobe var. pelopia Borkhausen, 1788	Current name: ***Fabriciananiobe* (Linnaeus, 1758)**
*Argynnislaodice* (Pallas, 1771)	***Argynnislaodice* (Pallas, 1771)**
Argynnislatoniavar.hungarica Aigner-Abafi, 1906	Current name: ***Issorialathonia*(Linnaeus, 1758)**
*Issorialathonia* (Linnaeus, 1758)	***Issorialathonia* (Linnaeus, 1758)**
*Brenthisino* Rottemburg, 1775	***Brenthisino* (Rottemburg, 1775)**
*Brenthisdaphne* ([Denis & Schiffermüller], 1775)	***Brenthisdaphne* ([Denis & Schiffermüller], 1775)**
*Brenthishecate* ([Denis & Schiffermüller], 1775)	***Brenthishecate* ([Denis & Schiffermüller], 1775)**
*Boloriaeuphrosyne* (Linnaeus, 1758)	***Boloriaeuphrosyne* (Linnaeus, 1758)**
*Boloriaselene* ([Denis & Schiffermüller], 1775)	***Boloriaselene* ([Denis & Schiffermüller], 1775)**
*Boloriadia* (Linnaeus, 1767)	***Boloriadia* (Linnaeus, 1767)**
Boloriapalessubsp.carpathomeridionalis ([Denis & Schiffermüller], 1775) Crosson & Popescu-Gorj, 1963	We mention that it is an endemic, accepted subspecies. ***Boloriapalescarpathomeridionalis* Crosson & Popescu-Gorj, 1963**
Inachis (Aglais) io (Linnaeus, 1758)	Current name: ***Aglaisio* (Linnaeus, 1758)**
*Aglaisurticae* (Linnaeus, 1758)	***Aglaisurticae* (Linnaeus, 1758)**
*Polygoniac-album* (Linnaeus, 1758)	***Polygoniac-album* (Linnaeus, 1758)**
*Polygoniaegea* (Cramer, 1775)	***Polygoniaegea* (Cramer, 1775)** The species is found in Eugen Worell’s collection and the specimen is not collected from Romania; it is also mentioned in the paper. **Material examined**: three specimens, June (without collection day), 1910, Pola Istrien (Croația), leg. Wo.
*Araschnialevana* (Linnaeus, 1758)	***Araschnialevana* (Linnaeus, 1758)**
Araschnialevanaf.porima Ochsenheimer, 1807	Current name: ***Araschnialevana* (Linnaeus, 1758)**
Araschnialevanaf.prorsa Linnaeus, 1758	Current name: ***Araschnialevana* (Linnaeus, 1758)**
Araschnialevanaf.intermedia Stichel, 1906	Current name: ***Araschnialevana* (Linnaeus, 1758)**
*Nymphalisantiopa* (Linnaeus, 1758)	***Nymphalisantiopa* (Linnaeus, 1758)**
*Nymphalispolychloros* (Linnaeus, 1758)	***Nymphalispolychloros* (Linnaeus, 1758)**
*Nymphalisxanthomelas* ([Denis & (Schiffermüller], 1775)	***Nymphalisxanthomelas* ([Denis & Schiffermüller], 1775)**
*Nymphalisvaualbum* ([Denis & Schiffermüller], 1775)	***Nymphalisvaualbum* ([Denis & Schiffermüller], 1775)**
*Euphydryasmaturna* (Linnaeus, 1758)	***Euphydryasmaturna* (Linnaeus, 1758)**
*Euphydryasorientalis* (Herrich-Schaffer, [1851])	It is not a European species. The species is distributed in Kazakhstan, Transcaucasia, south of the Ural Mountains.
*Euphydryasaurinia* (Rottemburg, 1775)	***Euphydryasaurinia* (Rottemburg, 1775)**
*Melitaeacinxia* (Linnaeus, 1758)	***Melitaeacinxia* (Linnaeus, 1758)**
*Melitaeaphoebe* ([Denis & Schiffermüller], 1775)	***Melitaeaphoebe* ([Denis & Schiffermüller], 1775)**
*Melitaeatrivia* ([Denis & Schiffermüller], 1775)	***Melitaeatrivia* ([Denis & Schiffermüller], 1775)**
*Melitaeadidyma* (Esper, 1778)	***Melitaeadidyma* (Esper, 1778)**
Melitaeadidymasubsp.alpina Staudinger, 1861	Current name: ***Melitaeadidyma* (Esper, 1778)**
Melitaeadidymasubsp.occidentalis Staudinger, 1861	The species is not collected from Romania. **Material examined**: two specimens, 23 July 1934, Kärnten/Karintia Ulrichsberg (Austria), leg. J. Thurner; 14 August 1962, Kärnten/Karintia Ulrichsberg 360 m (Austria), leg. J. Thurner.
Melitaeadidymasubsp.meridionalis Staudinger, 1870	Current name: ***Melitaeadidyma* (Esper, 1778)**
*Melitaeadidymoides* Eversmann, 1847	*Meliteadidymoides* is widespread in the Far East (eastern Siberia) not in Europe. Absent from the list of European Butterflies (Wiemers et al. 2018).
*Melitaeadictynna* Esper, 1778	Current name: ***Melitaeadiamina* (Lang, 1789)**
*Melitaeaaurelia* Nickerl, 1850	***Melitaeaaurelia* Nickerl, 1850**
Melitaeaparthenievar.varia Meyer-Dűr, 1851	The species is not collected from Romania. **Material examined**: two specimens, 18 July 1927, Tirol Rotmoostal (Austria), leg. O. Holik; 14 July 1930, Tirol Gaisbergatal (Austria), leg. O. Holik; We will mention that it is currently called: ***Melitaeavaria Herrich-Schaffer, 1851***
*Melitaeaathalia* (Rottemburg, 1775)	***Melitaeaathalia* (Rottemburg, 1775)**
Melitaeaathaliasubsp.dictynnoides Hormuzaki, 1898	Current name: ***Melitaeaathalia* (Rottemburg, 1775)**
*Melitaearetyezatica* Diöszeghy, 1930	**Material examined**: nine specimens, 26 July 1919/two specimens, Retezat Mountains (southern Carpathians), 950 m, leg. Diöszeghy; 28 July 1920, Retezat Mountains (southern Carpathians), 1600 m, leg. Diöszeghy; 4 July 1922, Retezat Mountains (southern Carpathians), leg. Diöszeghy; 7 June 1922/five specimens, Retezat Mountains (southern Carpathians), leg. Diöszeghy. Current name: **Melitaeaathaliaf.retyezatica (Diószeghy, 1930) (Urbahn and Zehdenick/Havel 1952)**
Melitaeaathaliasubsp.mehadiensis Gerhard, 1822	Current name: ***Melitaeaathalia* (Rottemburg, 1775)**
*Melitaeabritomartis* Assmann, 1847	***Melitaeabritomartis* Assmann, 1847**
Melitaeadiaminasubsp.alpestris Fruhstorfer, 1917	In Romania, we only have: ***Melitaeadiamina* (Lang, 1789)**
*Melitaeanana* Rehfons, 1910	Collection of Daniel Czekelius. Undecipherable labels. **Material examined**: two specimens, 25 July (no collecting year), Lands (?), leg. Cz; (no day and no month for collecting) ***Melitaeatrivia*** var. ***nana*** Staudinger, 1871
*Melitaeaparthenoides* Keferstein, 1851	This species has never been reported in Romania's fauna.
*Melitaeaasteria* Freyer, 1828	Erroneous determination in the collection of Eugen Worell. This species has never been reported in Romania's fauna.
*Limenitispopuli* (Linnaeus, 1758)	***Limenitispopuli* (Linnaeus, 1758)**
*Limenitiscamilla* (Linnaeus, 1764)	***Limenitiscamilla* (Linnaeus, 1764)**
*Limenitis sibilla (camilla)* Linnaeus, 1764	It is synonymous with: ***Limenitiscamilla* (Linnaeus, 1764)**
Limenitisreductasubsp.reducta Staudinger, 1901	Current name: ***Limenitisreducta* Staudinger, 1901**
*Neptisrivularis* (Scopoli, 1763)	***Neptisrivularis* (Scopoli, 1763)**
Neptisrivularissubsp.ludmila Nordmann, 1851	Current name: ***Neptisrivularis* (Scopoli, 1763)**
*Neptislucilla* ([Denis & Schiffermüller], 1775)	It is synonymous with: ***Neptisrivularis* (Scopoli, 1763)**
*Neptissappho* Pallas, 1771	***Neptissappho* (Pallas, 1771)**
*Neptisaceris* sensu Lhomme, 1924	It is synonymous with: ***Neptissappho* (Pallas, 1771)**
*Apaturailia* ([Denis & Schiffermüller], 1775)	***Apaturailia* ([Denis & Schiffermüller], 1775)**
Apaturailiavar.eos Rossi, 1794	Current name: ***Apaturailia* ([Denis & Schiffermüller], 1775)**
*Apaturaclytie* ([Denis & Schiffermüller], 1775)	Current name: ***Apaturailia* ([Denis & Schiffermüller], 1775)**
*Apaturairis* (Linnaeus, 1758)	***Apaturairis* (Linnaeus, 1758)**
*Apaturametis* Freyer, 1829	***Apaturametis* Freyer, 1829**
